# [^18^F]FDG PET/CT in Patients Affected by SARS-CoV-2 and Lymphoproliferative Disorders and Treated with Tocilizumab

**DOI:** 10.3390/jpm12111839

**Published:** 2022-11-04

**Authors:** Alberto Signore, Chiara Lauri, Maria Paola Bianchi, Sabrina Pelliccia, Andrea Lenza, Simone Tetti, Maria Luisa Martini, Gabriele Franchi, Fabio Trapasso, Luciano De Biase, Antonio Aceti, Agostino Tafuri

**Affiliations:** 1 Nuclear Medicine Unit, Department of Medical-Surgical Sciences and of Translational Medicine, Faculty of Medicine and Psychology, Sapienza University of Rome, 00185 Rome, Italy; 2 Haematology, “Sant’Andrea” University Hospital, Sapienza University of Rome, 00189 Rome, Italy; 3 Nuclear Medicine Unit, AOU Sant’Andrea, 00189 Rome, Italy; 4 Heart Failure Unit, Department of Clinical and Molecular Medicine, Sapienza University of Rome, 00189 Rome, Italy; 5 Infection Unit, Department NESMOS, Sapienza University of Rome, 00185 Rome, Italy

**Keywords:** COVID-19, pneumonia, [^18^F]FDG PET/CT, tocilizumab, SARS-CoV-2

## Abstract

Objectives: Interstitial pneumonia is a severe complication induced by the severe acute respiratory syndrome coronavirus 2 (SARS-CoV-2) infection. Several treatments have been proposed alone or, more often, in combination, depending, also, on the presence of other organ disfunction. The most frequently related, well-described, and associated phenomenon is pan-lymphopenia with circulating, high levels of cytokines. We report, here, on two patients with COVID-19 and lymphoproliferative disorders treated with Tocilizumab (a humanized monoclonal antibody against the interleukin-6 receptor) and followed by an [^18^F]FDG PET/CT to early evaluate the therapy’s efficacy. Methods: One patient with angioimmunoblastic T-lymphoma (A), one with Hodgkin lymphoma (A), and both with positive RT-PCR for SARS-CoV-2 and with similar clinical findings of interstitial pneumonia at the CT scan, were imaged by [^18^F]FDG PET/CT before and 14 days after a single dose of Tocilizumab. Results: In both patients, the basal [^18^F]FDG PET/CT showed a diffused lung parenchyma uptake, corresponding to the hyperdense areas at the CT scan. After 2 weeks of a Tocilizumab infusion, patient B had an improvement of symptoms, with normalization of the [^18^F]FDG uptake. By contrast, patient A, who was still symptomatic, showed a persisting and abnormal distribution of [^18^F]FDG. Interestingly, both patients showed a low bone marrow uptake of [^18^F]FDG at the diagnosis and after 15 days, while the spleen uptake was low only in lymphopenic patient A; both are indirect signs of an immune deficiency. Conclusions: In conclusion, in these two patients, interstitial pneumonia was efficiently treated with Tocilizumab, as demonstrated by the [^18^F]FDG PET/CT. Our results confirm that interleukin-6 (IL6) has a role in the COVID-19 disease and that anti-cytokine treatment can also be performed in patients with lymphoproliferative disorders.

## 1. Introduction

The coronavirus disease of 2019 (COVID-19) is a severe and often fatal syndrome that emerged in December 2019 in Wuhan, China. Infected patients may develop an excessive secondary immune response characterized by an uncontrolled pro-inflammatory cytokine and chemokine release known as a “cytokine storm” [1], which is sustained by the production of several pro-inflammatory cytokines and, in particular, interleukin-6 (IL-6), which correlates with the severity of the disease [2].

For therapeutic purposes, the use of the monoclonal antibody (MoAb), Tocilizumab, directed against the IL-6 receptor, is under investigation, and it has shown promising results in preliminary clinical studies [3,4,5]. Despite the role of [^18^F]FDG-PET/CT in COVID-19 being well-known [6,7], and also papers that have described the use of Tocilizumab for the treatment of COVID-19 [8,9,10], here we aimed at evaluating whether [^18^F]FDG-PET/CT can be useful in patients with COVID-19 and lymphoproliferative disorders treated with Tocilizumab.

## 2. Materials and Methods

### 2.1. Patients

In March 2020, two male patients were admitted to the emergency department of Sant’Andrea Hospital in Rome.

Patient A, 58 years old, had enlarged palpable lymph nodes of the neck associated with fever, weight loss, and an increased number of neutrophils (10.7 × 10^9^/L). The lymph node biopsy showed an angioimmunoblastic T-lymphoma.

Patient B, 68 years old, had a fever, night sweats, weight loss, enlarged palpable lateral-cervical lymph nodes, and anemia (Hb 7.8 g/dL). The lymph node biopsy revealed a classical Hodgkin lymphoma with mixed cellularity.

A nasopharyngeal swab for an RT-PCR for SARS-CoV-2 resulted in a positive for both patients.

### 2.2. Treatment and Imaging

Patients were immediately treated at that time with hydroxychloroquine, azithromycin, and methyl-prednisolone. Both patients were enrolled in the phase II trial of TOCIVID19 and received a single dose of intravenous Tocilizumab (8 mg/Kg). Before the Tocilizumab infusion, and again 2 weeks after the infusion, patients received a chest, high-resolution multilayer spiral CT scan (HRCT, 128 layers with Discovery, GE Healthcare, Boston, MA, USA) without contrast and an [^18^F]FDG PET/CT scan using a hybrid ToF PET/CT system (Biograph Horizon, Siemens, Munich, Germany) with a standard dose of [^18^F]FDG (4–5 MBq/Kg). PET images were acquired 60 ± 5 min after the [^18^F]FDG injection for 2.5 min per bed position with low-dose CT scans for the attenuation correction (140 kV, 90 mA, 0.8/s tube rotation, 5 mm thickness). Patients also performed a total body CT scan with contrast at the time of diagnosis for staging purposes.

Positivity to the [^18^F]FDG PET/CT was defined as the presence of at least one abnormal area of focal [^18^F]FDG uptake that was other than the physiological distribution or higher than the surrounding physiological tissue activity. Mean standardized uptake values (SUV_mean_) were calculated on axial sections by using 3 circular regions of interest (ROI) drawn in the upper, middle, and lower, as well as the left and right, lung, liver, thoracic aorta, bone marrow (D6, D9, and D12), and spleen [11,12]. Then, organ SUV_mean_/aorta SUV_mean_ ratios were calculated considering the aorta as background blood pool activity.

## 3. Results

### 3.1. Patients, Treatment, and Clinical Follow-Up

The treatment was well-tolerated without any side effects. The fever disappeared on day 3 after the Tocilizumab infusion. Clinical conditions rapidly improved in patient B and more slowly in Patient A, thus demonstrating a different therapeutic response.

Patient A: Two weeks after the Tocilizumab infusion, the patient showed improvement in their oxygen saturation and symptoms but still required symptomatic therapy with the addition of colistin and amphotericin B administration for an opportunistic infection of Acinetobacter Baumanii. At the end of this study, the patient started CHOP chemotherapy (cyclophosphamide, doxorubicin, vincristine, and prednisone) and recovered completely after 6 months.

Patient B: Two weeks after the Tocilizumab infusion, the patient showed improvement in their chest CT and PET/CT scans, despite the persistence of the SARS-CoV-2 infection, which was tested for by a pharyngeal swab and RT-PCR detection of the genome (RNA) SARS-CoV-2 virus. The patient started chemotherapy with an AVD regimen (doxorubicin, vinblastine, and dacarbazine) and recovered completely after 4 months.

Both patients were discharged after a double negative nasopharyngeal swab for SARS-CoV-2, having high levels of IgG and low levels of IgM anti-SARS-CoV-2.

At the 1-year follow-up, both patients were in complete remission and without medication.

The haematological data performed at the basal time and 2 weeks after therapy are reported in Table 1.

### 3.2. Chest HRCT and [^18^F]FDG PET/CT Scans

Patient A: The patient had a basal lung HRCT showing multiple and bilateral ground glass opacities that were widely organized in the pulmonary apices and associated with parenchymal consolidations prevalently distributed in the sub-pleural regions and pulmonary thickenings of the inter- and intra-lobular septa (a crazy paving pattern), predominantly located at the basis of the lungs. In addition, a whole-body CT showed enlarged lymph nodes (with a max diameter of 4 cm) partially conglobated in several node stations (the neck, mediastinum, and multiple abdominal regions).

**Figure 1:**
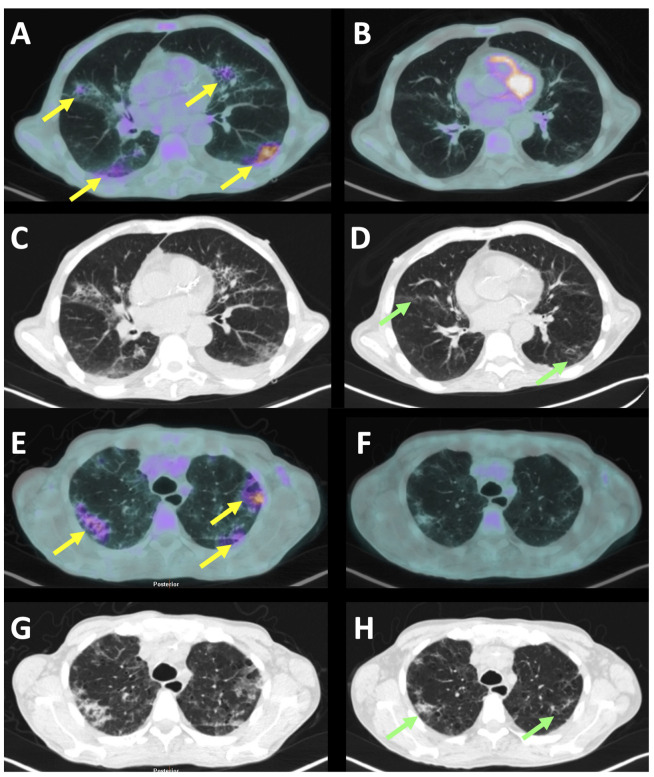
Axial section of the [^18^F]FDG-PET/CT scan of patient A (**A**–**D**) and patient B (**E**–**H**) before the Tocilizumab infusion (left images) and 14 days after therapy (right images). Yellow arrows indicate the areas of interstitial pneumonia that were metabolically active at the FDG-PET. After therapy, despite the FDG-PET being completely negative, there were still some areas of ground-glass at the CT scan (green arrows). Indeed, the normalization of the CT scan always occurred later than the normalization of the FDG-PET, thus highlighting the importance of monitoring patients’ therapy by FDG-PET.

The basal PET/CT showed a pathologic uptake in the upper-diaphragmatic lymph nodes (the neck, mediastinum, and supraclavicular regions) and an increased spleen volume with a normal hepato-splenic metabolic gradient. The lower-diaphragmatic lymph nodes, detected by the CT scan, were not characterized by an increased [^18^F]FDG uptake. In both lungs, an increased and diffused [^18^F]FDG uptake was observed, corresponding to the ground glass opacities at the CT scan and, in particular, in both posterior-basal regions of the lungs (Figure 1A–D). Two weeks after the Tocilizumab, a PET/CT showed a modest improvement of both the haematological and chest diseases, with a persisting residual [^18^F]FDG uptake of lower intensity, compared to that of the basal study, in both lungs and the upper-diaphragmatic lymph nodes.

Patient B: The patient had a basal lung HRCT scan showing bilateral and diffused ground-glass opacities prevalently distributed in the sub-pleural regions with evident fibrous thickenings of the inter- and intra-lobular septa (a crazy paving pattern). A whole-body CT showed multiple and partially conglobated lymph nodes at the para-esophageal site, at the level of the celiac tripod, in the small gastric curvature, in the para-aortic area, in the inter-portal-cava region, and at the hepatic hilum, and showed an increased spleen and liver volume. A follow-up HRCT scan of both patients showed an improvement in pneumonia, with a significant reduction of the ground glass areas and a residual “crazy paving pattern”.

The basal PET/CT showed a pathologic uptake in many of the lymph nodes of the upper- and lower-diaphragmatic stations (the neck, mediastinum, left axillary region, supraclavicular regions, para-esophageal site, celiac, peri-gastric curvature, in the para-aortic area, inter-portal-cava region, and at the hepatic hilum) and an increased spleen volume with an inversion of the hepato-splenic metabolic gradient. In both lungs, a slightly increased and diffused [^18^F]FDG uptake was observed, corresponding to the ground glass opacities at the CT scan and, in particular, in both the posterior-basal regions of the lungs (Figure 1E–H).

A follow-up study after the Tocilizumab showed a progression of the haematologic disease and complete normalization of the [^18^F]FDG uptake in the lungs.

The SUV_mean_ in the tissues are reported in Table 2. Interestingly, despite patient A being lymphopenic (at the baseline and after 14 days) and patient B having normal white blood cells, the liver and spleen uptake of [^18^F]FDG (the liver/aorta and spleen/aorta ratios) increased in both patients during the follow-up (Table 2). By contrast, the bone marrow activity improved only in patient B but remained low in lymphopenic patient A.

## 4. Discussion

Recently, several drugs have been proposed for treating patients with COVID-19 interstitial pneumonia and lymphopenia. Amongst these is Tocilizumab, an immunosuppressive drug used for the treatment of rheumatic disorders in adults and children. It is a humanized monoclonal antibody against the IL6 receptor (IL6R).

In March 2020, Perrone et al. suggested the use of Tocilizumab to modulate the cytokine cascade and cytokine storm in patients with COVID-19 [13]. At the same time, others tested the treatment of COVID-19 with Tocilizumab [14]. Based on promising results, Roche and the WHO launched separate trials for its use in severe COVID-19 [15].

In our two patients, the choice to start Tocilizumab was made not only for COVID-19 pneumonia but also, and most importantly, because of the symptoms of the haematological disease and high levels of IL-6 in the serum (135.0 pg/mL for patient A and 187.0 pg/mL for patient B; normal values < 1.8 pg/mL). Tocilizumab improved COVID-19 pneumonia, as shown by the follow-up PET/CT scan, and reduced the haematological symptoms. This treatment also probably contributed to the avoidance of the progression of the haematological disease in Patient A, which remained stable.

Patient B, two weeks after the Tocilizumab infusion, started chemotherapy with an AVD regimen with a reduced dose. To the best of our knowledge, there is only one case reported of Hodgkin lymphoma in complete remission affected by COVID-19 and successfully treated with Tocilizumab due to interstitial pneumonia [3].

The role of [^18^F]FDG PET/CT for both the diagnosis and therapy follow-up of haematological disorders is now well-consolidated, as well as for the diagnosis and therapy assessment of infections and inflammations [16,17]. However, to the best of our knowledge, no papers have investigated the potential application of this modality for the early evaluation of the treatment’s efficacy in COVID-19 patients. Furthermore, [^18^F]FDG PET/CT is useful for the assessment of the metabolic status of all organs, such as the bone marrow, spleen, and gut, which may be involved in the development and progression of a cytokine storm or haematological disease. Indeed, as for other infective diseases, autoimmune diseases, and lymphoproliferative diseases [11,12], we were also expecting hypermetabolism of the spleen in COVID-19 patients, as demonstrated by others in convalescing COVID-19 patients [18].

On the contrary, in our patients, the activity in the spleen was rather low or normal both at the time of diagnosis and after 14 days, and in patient A, we also found a low FDG uptake in the bone marrow.

In the literature, we found only five case reports clearly showing hypometabolism of the spleen in two patients, one with Hodgkin lymphoma [19] and one with Large B-cell lymphoma [20], and hypermetabolism of the spleen in two patients, one with Mantle Cell lymphoma [21] and one with non-Hodgkin lymphoma [22]. Finally, a patient with Hodgkin lymphoma clearly showed hypermetabolism of both the spleen and bone marrow [23]. Therefore, in patients with concomitant lymphoproliferative disorders and COVID-19, [^18^F]FDG may show an atypical organ distribution that deserves further investigation.

In conclusion, our data support what emerges from the literature; that [^18^F]FDG-PET/CT should be used in the follow-up of COVID-19 patients to evaluate the response to treatments. The role of the spleen and bone marrow in lymphopenia remains to be evaluated, although recent studies have suggested the role of the large bowel in lymphocyte margination [24].

Although we cannot provide direct evidence that Tocilizumab treatment is useful in patients with COVID-19 and lymphomas, our results confirm that IL-6 plays a role in the COVID-19 disease and anti-cytokine treatment should be further explored.

## Figures and Tables

**Table 1:** Haematological data in patients before and 14 days after Tocilizumab.

	Patient A		Patient B	
	At Diagnosis	After Therapy	At Diagnosis	After Therapy
**WBC (cells × 10^9^/L)**	3310	6520	3200	4040
**RBC (cells × 10^9^/L)**	3,000,000	3,300,000	2,400,000	3,200,000
**Platelets (cells × 10^9^/L)**	124,000	130,000	56,010	159,000
**Lymphocytes (cells × 10^9^/L)**	360	510	840	500

**Table 2:** Values of SUV_mean_ in different tissues in the patients before and 14 days after the Tocilizumab, as compared to the control subjects.

	Patient A		Patient B		Normal Subjects (*n* = 18)
	At Diagnosis	After Therapy	At Diagnosis	After Therapy	Mean ± SD (Min-Max)
**Liver**	2.56	2.50	2.12	1.99	2.49 ± 0.34 (1.89–3.07)
**Spleen**	2.07	1.75	2.37	2.06	2.03 ± 0.26 (1.49–2.95)
**Bone marrow**	2.08	1.65	1.87	1.86	1.78 ± 0.23 (1.20–2.44)
**Thoracic aorta**	1.93	1.56	1.66	1.32 *	1.94 ± 0.28 (1.51–2.42)
**Liver/Aorta**	1.33	1.60	1.28	1.51	1.30 ± 0.21 (0.92–1.74)
**Spleen/Aorta**	1.07	1.12	1.43 *	1.56 *	1.07 ± 0.14 (0.90–1.38)
**Bone marrow/Aorta**	1.08	1.06	1.13	1.41 *	0.95 ± 0.20 (0.56–1.25)
**Spleen/Liver**	0.80	0.70 *	1.11	1.03	0.84 ± 0.14 (0.71–1.25)

* Values in bold are out of the normal range.

## Data Availability

Data are available upon request from the corresponding author.
